# Adaptive Evolution of Rhizobial Symbiosis beyond Horizontal Gene Transfer: From Genome Innovation to Regulation Reconstruction

**DOI:** 10.3390/genes14020274

**Published:** 2023-01-20

**Authors:** Sheng Liu, Jian Jiao, Chang-Fu Tian

**Affiliations:** 1 State Key Laboratory of Plant Environmental Resilience, College of Biological Sciences, China Agricultural University, Beijing 100193, China; 2 MOA Key Laboratory of Soil Microbiology, Rhizobium Research Center, China Agricultural University, Beijing 100193, China

**Keywords:** rhizobia, adaptive evolution, symbiosis, nitrogen fixation, nodulation

## Abstract

There are ubiquitous variations in symbiotic performance of different rhizobial strains associated with the same legume host in agricultural practices. This is due to polymorphisms of symbiosis genes and/or largely unexplored variations in integration efficiency of symbiotic function. Here, we reviewed cumulative evidence on integration mechanisms of symbiosis genes. Experimental evolution, in concert with reverse genetic studies based on pangenomics, suggests that gain of the same circuit of key symbiosis genes through horizontal gene transfer is necessary but sometimes insufficient for bacteria to establish an effective symbiosis with legumes. An intact genomic background of the recipient may not support the proper expression or functioning of newly acquired key symbiosis genes. Further adaptive evolution, through genome innovation and reconstruction of regulation networks, may confer the recipient of nascent nodulation and nitrogen fixation ability. Other accessory genes, either co-transferred with key symbiosis genes or stochastically transferred, may provide the recipient with additional adaptability in ever-fluctuating host and soil niches. Successful integrations of these accessory genes with the rewired core network, regarding both symbiotic and edaphic fitness, can optimize symbiotic efficiency in various natural and agricultural ecosystems. This progress also sheds light on the development of elite rhizobial inoculants using synthetic biology procedures.

## 1. Introduction

Rhizobia refer to a polyphyletic group of Gram-negative bacteria that induce nodule formation on roots, or occasionally stems, of leguminous plants, where they reduce N_2_ into ammonia [1]. Rhizobia live in soils saprophytically and, under suitable conditions, enter into an intracellular symbiotic relationship with compatible plant hosts, which requires a multi-step molecular “dialogue” between symbiotic partners during rhizoplane colonization, infection, nodule organogenesis and senescence [2,3]. This represents a typical facultative lifecycle of microsymbionts [4]. Within the infected legume nodule cells, rhizobia proliferate and then undergo either terminal or non-terminal differentiation into nitrogen-fixing bacteroids, which are surrounded by plant-derived membrane to form the organelle-like symbiosome [5]. The terminal differentiation due to irreversible loss of cell division ability is typically initiated by nodule-specific cysteine-rich (NCR) peptides from host legume species of the Inverted Repeat Lacking Clade (IRLC, e.g., alfalfa and pea) and *Aeschynomene* [6,7,8,9]. This terminal differentiation phenomenon is however not observed for bacteroids in other legumes, e.g., soybean and common bean [10]. Legume hosts provide carbon and other essential resources for bacteroids to support the energetically expensive but O_2_-sensitive nitrogen-fixing reaction [2]. To allow respiration of bacteroids but avoid nitrogenase inactivation, free O_2_ in host cells is modulated by leghemoglobins as low as ~50 nM [11,12,13,14]. These distinct features support the rhizobium–legume symbiotic nitrogen fixation (SNF) as the most efficient biological nitrogen fixation system in nature. About 40 million tons of N are fixed by the rhizobium–legume SNF per year, accounting for about 65% of the total input of biological nitrogen fixation in agricultural systems [15]. Meanwhile, many legume species, e.g., soybean, common bean and alfalfa, are important food and forage crops, globally contributing to the sustainable agriculture.

Rhizobial SNF is an adaptive trait evolved in a subset of free-living soil bacteria. This symbiotic trait is mainly attributable to key nodulation and nitrogen fixation genes [16,17], which are generally clustered as two key gene circuits on symbiosis islands or symbiosis plasmids [18,19]. These key symbiosis genes have been found in more than 200 validly published rhizobium species, belonging to 18 genera of *Alpha-* and *Betaproteobacteria* [1], except some *Bradyrhizobium* strains that can establish symbiosis with certain *Aeschynomene* species or soybean in *nod*-independent ways [20,21,22]. The most recent common ancestor of *Rhizobiales* members did not possess key symbiosis genes [23], and *Alpha-* and *Betaproteobacteria* are over-represented in the core root microbiome of terrestrial plants [24,25]. Cumulative evidence based on molecular phylogenies and comparative genomics [23,26,27,28] supports the multiple origin hypothesis for rhizobia: predisposed soil bacteria with different taxonomic affiliations have evolved to rhizobia by obtaining key symbiosis genes repeatedly and independently [16,29].

**Figure 1:**
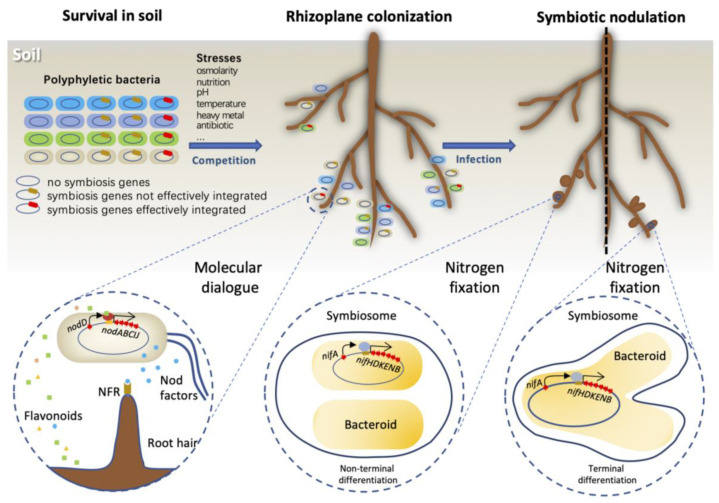
Adaptive evolution of rhizobium symbiosis. After acquiring the key symbiosis genes (*nod* and *nif*) by horizontal gene transfer (HGT), bacteria recipients confront ever-fluctuating environmental conditions and resources, and interactions with other soil microorganisms and plants. During the process of adaptive evolution, those bacteria, with the key symbiosis genes effectively integrated with the genomic background, survive the saprophytic life, colonize the host rhizoplane, communicate and establish a mutualistic symbiosis relationship with the host plant. In a strain–host-dependent manner, the nitrogen-fixing rhizobia, named bacteroids, are at either non-terminal or terminal differentiation status within symbiosome. *nod*, nodulation; *nif*, nitrogen fixation; NFR, nodulation factor receptor.

Despite the polyphyletic diversity pattern of rhizobia, obtaining key symbiosis genes through horizontal gene transfer (HGT) does not guarantee the recipient bacterium an effective symbiotic ability [30,31]. Introducing key symbiosis gene circuits into non-rhizobia, e.g., *Agrobacterium tumefaciens*, *Escherichia coli* and *Ralstonia solanacearum*, confers on these recipients the ability of inducing nodule-like structures on test legumes [30,31], or even morphologically normal and infected nodules after selection in an experimental evolution screen on *R. solanacearum* derivatives [31]. However, these evolved strains are still not capable of fixing nitrogen [30,31,32,33]. By contrast, clones of nascent nitrogen fixation ability are more likely to evolve, under both field and laboratory conditions, from soil bacteria belonging to typical rhizobial genera, e.g., *Mesorhizobium* and *Sinorhizobium* [34,35,36,37,38]. These findings suggest a strong recipient-dependent effect. It is noteworthy that there are also extensive efficiency variations in symbiotic interactions between closely related rhizobia associated with the same legume under both controlled and fluctuating field conditions, which significantly affect the inoculation efficiency of rhizobial inoculants in agriculture practices [39,40]. For example, some indigenous rhizobia with low efficiency of nitrogen fixation show superior local fitness, which challenged the performance of introduced commercial rhizobium inoculants [34,39,41]. An elite rhizobial inoculant needs to fulfil at least the following criteria: (1) survival under fluctuating edaphic conditions; (2) efficient rhizoplane colonization; (3) successful integration of symbiosis genes and compatible symbiotic interactions with hosts (Figure 1). This review aimed to summarize our current understanding of rhizobial adaptive evolution, particularly on genome innovations and network rewiring processes, underlying the successful integration of key symbiosis circuits in diverse bacteria.

## 2. Genome Innovations after Receiving Key Symbiosis Genes

**Figure 2:**
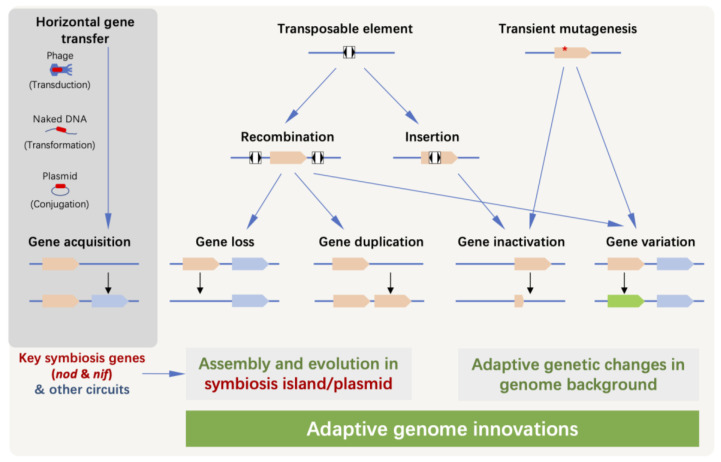
Genome innovations after receiving key symbiosis genes. The main patterns of rhizobial genome innovation in adaptive evolution include gene acquisition, loss, duplication, inactivation and variation, mediated by transposable elements and transient hyper-mutagenesis. Red star indicates a point mutation.

Although the relative importance of different DNA uptake mechanisms (transduction, transformation and conjugation; Figure 2) in acquiring key symbiosis genes is rarely investigated in rhizobia, genetic diversity of field isolates and experimental evolution studies provide strong evidence for HGT of key symbiosis genes [34,35,36,37,38]. The recipient-dependent integration efficiency of key symbiosis genes is consistent with that the evolution of any trait is an individual innovation constrained by ancestral conserved traits. At the genomic level, adaptive evolution is a process in which heritable variability that arises constantly from the interaction between organisms and the *n*-dimensional niche is selected and fixed [42,43,44]. According to the infinitesimal model of evolution, adaptation is driven by the accumulation of numerous mutations, and each one has an infinitesimal influence, highlighting the genetic complexity of adaptive traits as quantitative [45]. Moreover, a large number of standing studies also emphasized that specific genes or loci may contribute to adaptation in qualitative ways in the context of adaptive evolution of pangenome [46,47]. Here, we summarize known evolutionary processes shaping genome innovations post receiving key symbiosis genes in model rhizobia (Figure 2).

### 2.1. Continuous Evolution of Key Symbiosis Genes

The common *nodABC* genes are responsible for the synthesis of N-acetylglucosamine oligosaccharide backbone of nodulation factors (NFs), which are secreted through NodIJ and determine host specificity; while *nifHDK* and *nifENB* are nitrogenase structural and FeMo-co biosynthesis genes, respectively [16,17]. These *nod* and *nif* genes, together with their transcriptional activator genes *nodD* and *nifA*, can be considered as key symbiosis genes providing nodulation and nitrogen fixation potential, respectively (Figure 1). The nodulation genes play a crucial role in the adaptation of NF-dependent rhizobia to their hosts, as the match between NFs and host NF receptors directly determines whether a symbiotic relationship can be initiated [3]. Homologs of canonical *nodABC* and *nodH*, involved in NF synthesis and modification, have been found in certain *Frankia* strains, which are more deeply rooted than those from rhizobia [48,49,50]. A *nodC* gene from *Frankia* Dg1 restores the ability of a *nodC* mutant of *Rhizobium leguminosarum* A34 to induce root hair deformation (but not nodulation) [48]. Paralogs of *nodIJ*, involved in secreting NFs in rhizobia, are only found in *Betaproteobacteria* but not in those rhizobia belonging to *Alphaproteobacteria* [51]. Homologs of NodD, sensing host signals and activating the transcription of nodulation genes, are present in all rhizobia including those lacking *nodABC* [20,52]. Therefore, it seems that the circuit of key nodulation genes had been naturally assembled through multiple events of gene duplication, vertical and horizontal evolution [53].

The extraordinary variety of legume species and hence the NF receptors likely select rhizobia producing compatible NFs out of those secreting NFs varying in the length and modifications of N-acetylglucosamine oligosaccharide backbone [54]. This view has been elegantly proved in a pioneer study where NodA from *Sinorhizobium meliloti* but not that from *Rhizobium tropici* can transfer unsaturated C16 fatty acids onto the NF backbone, leading to host specificity [55]. NodC determines the length of NF backbone [56], and variations in *nodC* among *S. meliloti* strains determine host specificity with different *Medicago* species [57]. This and similar evidence supports a rational proposal of rhizobial “symbiovar” that is distinguished by the host range and corresponding variation of key symbiosis genes, e.g., *nodA* and *nodC* that have been widely used in rhizobial diversity studies [58,59].

Notably, different cocktails of NFs can be secreted by the same strain under fluctuating conditions, e.g., pH [60], but the underlying mechanisms remain elusive. Three distinct *nodA* genes are present in two broad-host-range strains *R. tropici* CIAT 899 and *Rhizobium* sp. PRF 81 [61]. Rhizobial NodD can specifically sense symbiotic signals out of the cocktail of legume root exudates e.g., flavonoids, and the number of *nodD* copies varies among rhizobia [62,63]. A NodD from the broad-host-range strain MPIK3030 is sensitive to a broader range of flavonoids than three NodDs of *S. meliloti* [64]. Functional differentiation between NodD copies in the same rhizobium, e.g., *S. meliloti*, *Sinorhizobium fredii*, *R. tropici* and *Bradyrhizobium diazoefficiens*, has been demonstrated [65,66]. Interestingly, different *nodD* combinations out of five copies in *R. tropici* CIAT 899 are required for nodulation on different hosts [67]. There is evidence showing recognition of cereal root exudates by NodD1 from the broad-host-range *S. fredii* NGR234 and subsequent induction of *nod* genes [68]. Ample evidence also demonstrates that different rhizobial species that nodulate the same plant can secrete NFs of different length and structures [54,69], and their nodulation genes, e.g., those from *Bradyrhizobium* and *Sinorhizobium* nodulating soybeans, show a genus-dependent evolutionary history [52,70,71]. Moreover, certain rhizobia show a broad-host-range nodulation ability not only due to their NF cocktails [72]. On the other hand, some photosynthetic *Bradyrhizobium* strains, lacking the canonical nodulation genes *nodABC*, have evolved an ability to induce nodules on some *Aeschynomene* species in an NF-independent way [20], while introducing a symbiosis plasmid carrying canonical nodulation genes can block this unique symbiosis [73].

These non-exhaustive examples support a continuous evolution model of the rhizobial nodulation gene circuit in various bacterial recipients, which at least involves gene divergence, gene duplication and/or loss (Figure 2). In contrast to the nodulation gene circuit, key genes (*nifHDKENB*) for producing nitrogenase and its FeMo-co show a much broader phyletic distribution in both bacteria and archaea. Recent molecular evolution analysis supports a bacteria-first hypothesis for the origin of nitrogen fixation genes [74]. In *Proteobacteria* including both rhizobia and non-symbiotic diazotrophs, key nitrogen fixation genes are directly activated by NifA [11]. Among rhizobia, the nitrogen fixation gene circuit can be horizontally transferred together with the nodulation gene circuit, as a symbiosis island or within a symbiosis plasmid, under both laboratory and field conditions [35,36]. However, key nitrogen fixation genes that are active in nodules can have close non-symbiotic homologs in the same genome of certain *Bradyrhizobium* strains or in non-rhizobial strains of the same genus [26,75]. Moreover, there is evidence showing HGT events with the nodulation gene circuit but not nitrogen fixation genes from *Azorhizobium caulinodans* to other bacteria [37]. Therefore, the evolutionary history of nodulation and nitrogen fixation gene circuits can be decoupled among diverse rhizobia.

### 2.2. Horizontal Transfer of Genes beyond Key Symbiosis Genes

There is no doubt that HGT of key symbiosis genes plays a dominant role in shaping rhizobial diversity, thus benefiting both bacterial recipients and associated plant hosts [16,17]. Available genomics analyses show that rhizobia have open pangenomes, indicating that HGT of accessory genes is extensive, while key symbiosis genes are only a small part of the accessory genome [52,76,77]. Among closely related rhizobial species/strains, many detectable HGT events involve plasmid-encoded genes [78,79,80]. For example, in an investigation into 196 *R. leguminosarum* sv. *trifolii* genomes belonging to a five-species complex, 171 genes including symbiosis genes were shown to have inter-species introgression, with symbiosis genes showing a distinct selection signature [78]. Notably, some introgression genes of the *fixNOQPGHIS* cluster exhibit multiple genomic locations and can be independent on symbiosis plasmid-associated introgression events [78]. Similarly, another or multiple *fixNOQPGHIS* copies, not localized on the symbiosis island/plasmid, can be found in many rhizobial genomes [34,81]. This is consistent with the fact that *fixNOQP* and *fixGHIS* are required for assembly and function of the *cbb3* high-affinity terminal oxidase under both free-living microaerobic and symbiotic conditions [81,82]. On the other hand, these findings also support that *fix* genes integrated into symbiosis island/plasmid may be an adaptive trait in terms of fast spreading of the symbiotic ability among soil bacteria. 

Among genes co-localized with key symbiosis genes of some broad-host-range rhizobia, those encoding type III secretion system (T3SS) and its effector proteins have received considerable attention and investigation. Different effector proteins or natural variation of the same effector can positively, neutrally, or negatively affect symbiotic compatibility depending on host genotypes [83,84,85,86]. A similar scenario fits with the type IV secretion system (T4SS). In certain *Mesorhizobium* strains lacking T3SS, host range change can be mediated by mutations in *vir* genes encoding the T4SS components or in genes encoding effector proteins [87]. The T4SS gene clusters are localized on the symbiosis island of *Mesorhizobium*, and the phylogeny of *traG*, encoding a substrate receptor of T4SS, is similar to that of key symbiosis genes (*nod* and *nif*) [88]. Moreover, there are multiple lines of evidence supporting coordinated transcriptional regulation of T3SS or T4SS genes and *nod* genes by NodD and legume root exudates [83,87,88,89,90]. The proximity of conditional beneficial genes and key symbiosis genes on symbiosis island/plasmid, and their co-transfer between different bacteria are evolutionarily meaningful, because they contribute to fast spreading of symbiotic functions, particularly when rhizobia are interacting with different legume hosts.

In rhizobia with multipartite genome architecture, the existence of accessory plasmids with conjugative transfer ability makes HGT easier to happen. Genome sequence analyses of 92 reference strains of *Rhizobiaceae* show that gene acquisition events related to accessory plasmids introduced more genes into the genomes of nitrogen-fixing species, which expanded the metabolic ability of rhizobial species and may facilitate the adaptation to various environmental conditions [91]. In *S. meliloti*, accessory plasmids are similar in many ways to the symbiosis plasmid, and HGT between these plasmids contributes to the gene content of the symbiosis plasmid and the constantly evolving symbiotic phenotypes [92]. A stress-tolerant alfalfa microsymbiont *S. meliloti* B401 has a highly similar genomic background with the model strain *S. meliloti* 1021, but with an additional set of genes encoding the uptake system for betaine and choline on the symbiosis plasmid, which might partially explain its high levels of SNF under both humid and semiarid environments [93]. Among tested *Sinorhizobium* strains showing different symbiotic performance on certain soybean cultivars, an elite strain has three accessory zinc transporter genes (*zip1*, *zip2* and *c06450* localized on the chromid or accessory plasmid) in addition to the conserved chromosomal *znuABC* (high affinity zinc transporter), which make cumulative contributions to the zinc homeostasis and nodulation compatibility [94].

Genes on accessory plasmids, in some cases, may only be beneficial to rhizobia but harmful to the plant, an evolutionary scenario similar to parasitism. For example, certain *Sinorhizobium* strains carry the *hrrP* gene on accessory plasmids, and HrrP degrades the host-derived NCR peptides, providing adaptability to the microsymbionts at the expense of the host fitness [95]. The transfer of certain accessory plasmids between *S. meliloti* strains enhances competitive nodulation ability of the recipient on alfalfa plants, but with reduced nitrogen fixation ability [96]. These cases suggest the transformation of beneficial rhizobia into a more exploitative way of life mediated by accessory genes on plasmids [95,96].

### 2.3. Gene Inactivation, Gene Loss and Genome Rearrangement in Symbiosis Plasmid/Islands

As facultative microorganisms, rhizobia face a very complex and fluctuating environment, and undergo a great ecological shift. The relatively large and plastic genome generally support the adaptability; however, some of the pre-existing genes or metabolic pathways may sometimes be harmful when they shift to a new habitat or lifestyle. In this case, loss or inactivation of the corresponding genes under purifying selection pressure can provide them with adaptive advantages. At the stage of host infection, some secreted effector proteins or virulence systems may trigger the immune response of certain hosts, thus affecting the cell entry. For example, T3SS and its effectors have been independently reported to be key factors limiting rhizobial compatibility during adaptive evolution under the selection of new hosts [31,84,85,97]. This can be mediated by transient hypermutability stage before infection due to a mutagenesis *imuABC* cassette on the symbiosis plasmid [98], and/or by insertion mutation and gene loss mediated by transposable insertion sequences (ISs) that are enriched on the symbiosis plasmid or symbiosis island [84,85,97]. 

Phyletic distribution analysis shows that *imuABC* genes are over-represented in rhizobia harboring symbiosis plasmids. The *imuABC* genes encode error-prone DNA polymerase, the expression of which can lead to high-frequency mutations [98]. In the experimental evolution studies conducted by the Masson-Boivin laboratory, when symbiosis genes were transferred together with the mutagenesis *imuABC* cassette, the mutation rate of the recipient genome increased and the evolution of soil bacteria to rhizobium accelerated [98]. Exposure to either plant culture medium or *Mimosa* plants triggered transient hyper-mutagenesis of the recipient bacteria. Adaptive mutations leading to major phenotypic changes were identified: stop mutations of structural T3SS component *hrcV* led to nodulation [31]; stop mutations in virulence regulatory factor *hrpG* or mutations in the promoter region or frameshift mutations in its upstream sigma factor *prhI* and *vsrA* genes led to primitive infection of nodule cells [32,98]. Missense mutations in the global regulatory factor *efpR*, or intergenic mutations in the upstream of unknown functional genes [33], or mutations in two different components of the Phc quorum sensing system, *phcB* or *phcQ*, lead to massive infection of nodule cells, separately [99]. Following saprophytic-symbiotic lifestyle shift guarantees the mutations beneficial to the plant host being preserved [17]. The *imuABC*-based transient mutagenesis, together with the following host selection, represent a two-step evolutionary scenario of rhizobial adaptation [100]. This is supported by the fact that both natural and experimental processes result in rapid genetic diversification dominated by purifying selection [100].

Transposable elements (TEs) were considered as the “junk” and “selfish” components of genome, while cumulative evidence suggests that they are important players in the evolution of both eukaryotes and prokaryotes [101]. TEs amplify to very high copy numbers when entering into a new niche, resulting in a large number of variations, making cellular organisms adapt to the new environment quickly [102]. The IS is the most common TE in bacteria. In addition to disrupting the gene by insertion, ISs can also promote gene inversion, deletion, duplication, and fusion of two replicons [103,104]. Most rhizobial genomes are rich in IS elements that are concentrated in packages of symbiosis genes. For example, in *S. fredii* NGR234, up to 18% of the symbiosis plasmid pNGR234a are mosaic sequences and ISs [105]; ISs are also abundant on the symbiosis plasmids of other *Sinorhizobium* strains [84,106], and on the 500-kb symbiosis island embedded on the chromosome of symbiotic *Mesorhizobium* and *Bradyrhizobium* strains [19,107,108]. By contrast, ISs are rarely present on the *nif* islands of most sequenced non-symbiotic *Bradyrhizobium* strains [109], implying a relatively limited evolutionary potential of key *nif* genes compared to those genes modulating symbiotic compatibility. Indeed, adaptive evolution of symbiotic compatibility due to gene insertion and loss in the T3SS gene cluster mediated by ISs have been observed in the experimental evolutionary studies of *Sinorhizobium* and *Bradyrhizobium* strains associated with soybeans [84,85,97]. Gene loss is mediated by homologous recombination of ISs [97], and insertion mutation rates are in line with a niche differentiation model of ISs [110]. In addition, gene duplication within the symbiosis island mediated by homologous recombination between IS copies also exists in nature [97].

The major active ISs, in the adaptive evolution of *Sinorhizobium* strains, display broader phyletic and replicon distribution than other ISs, and prefer target sequences with AT-rich content, which is a characteristic feature of symbiosis plasmids [84]. Recently, such biased distribution and insertion rates of ISs are experimentally demonstrated by using an intracellular “common garden” approach adapted from conventional ecology [110]. In this genome ecology experiment, conditional lethal *sacB* gene of low, medium or high GC content was individually inserted into three replicons of a model bacterium *S. fredii* to trap transposable ISs in the process of adaptive evolution [110]. Xenogeneic *sacB* of low and medium GC% in the low GC% symbiosis plasmid are preferred by major active ISs, and such preference is dependent on MucR, a conserved xenogeneic silencer in *Alphaproteobacteria* [111,112]. This is at least partially due to MucR also preferring to target AT-rich DNA sequences of the symbiosis plasmid and possessing a DNA-bridging ability that may facilitate transposition [111,112]. Notably, in addition to other copies, *mucR* can be found on the symbiosis plasmid or other transferable genomic regions [113]. The processes mentioned above mediated by ISs and MucR, together with the *imuABC*-dependent hypermutations, may shape the gene content, gene order and genetic variation of the constantly evolving symbiosis gene circuits on symbiosis island/plasmid, contributing to the fast adaptive evolution of rhizobial symbiotic compatibility. Particularly, genome rearrangement in the symbiosis plasmid mediated by ISs may facilitate the assembly of key symbiosis gene circuits with new symbiotic players, which further supports the innovation and fast spreading of symbiotic function to other soil bacteria. These molecular evolutionary mechanisms can also help people better understand, modify and utilize these genome modification tools, so as to promote the domestication or genetic stability of inoculants.

## 3. Reconstruction of Regulatory Networks

The rhizobium symbiosis involves biological processes including communication with plant host, migration to the rhizosphere, rhizoplane colonization, induction of nodule and infection thread, intracellular host infection, accommodation in the plant cell, morphological differentiation, lifestyle change and cell function specialization [2]. This represents a typical complicated trait that needs not only key symbiosis genes but also a large number of core and lineage-specific functions [16,52,114,115]. Together with scattered genetic evidence, recent advances in high-throughput analyses of rhizobial fitness genes, metabolic and regulation networks during lifestyle adaptations, are providing more insight into the integration mechanisms of symbiotic function within bacterial recipients.

### 3.1. Recruitment of Indigenous Functions to Support Symbiosis

Like any bacteria-plant interactions, the rhizobium–legume symbiosis is a complex and delicate process, which involves the cooperation of multiple cellular functions. Metabolic modeling of *S. meliloti* suggests that chromid genes are more actively involved in rhizosphere fitness than in bulk soils, while chromosome has a similar contribution to fitness in two niches [116,117]. A large number of *R. leguminosarum* genes are differentially expressed in rhizospheres of pea, alfalfa and sugar beet [118]. Some host-specific genes are related to C metabolism, and many are located on the non-symbiosis accessory plasmids [118]. These findings indicate that non-symbiosis genes are extensively involved in rhizosphere fitness. By using the transposon insertion sequencing method, about 600 genes of *R. leguminosarum* are identified as fitness genes during lifestyle adaptations from rhizosphere to symbiosis with pea plants [115]. Comparative transcriptomics independently demonstrates that hundreds of rhizobial genes are differentially expressed in nodules compared to free-living cells [114,119,120,121,122]. Metabolic modeling suggests global coordination of carbon and nitrogen allocation in bacteroids [123]. The broad-host-range strain *S. fredii* NGR234 shows considerable differences in transcriptomes of bacteroids in *Leucaena leucocephala* and *Vigna unguiculata* [121]. Key nitrogen fixation genes on the symbiosis plasmid of *S. fredii* strains in *Glycine* nodules are characterized by high connectivity in both intra- and inter-replicon co-expression analyses [114], which allow further identification of chromosomal core *znu* and accessory *mdt* operons involved in host-specific symbiotic adaptation [114]. However, functional characterizations post genome-wide surveys are usually limited [114,115].

Based on scattered genetic and molecular biological evidence, a general picture of extensive recruitment of core and accessory functions, in addition to key nodulation and nitrogen fixation genes, in optimizing symbiotic efficiency has been proposed earlier [16,17]. The list of core and accessory functions contributing to rhizobial fitness, from rhizosphere to rhizoplane to nodules, is ever-increasing (above 900 genes) and usually in a strain–host-dependent manner [52], e.g., motility and chemotaxis [124], surface polysaccharides [125,126,127,128,129], outer membrane vesicles [130], quorum sensing [131,132,133,134,135], T1SS, T3SS, T4SS and T6SS [136,137,138,139,140,141,142], dicarboxylate transport [143], poly-3-hydroxybutyrate [144,145,146], transporters of branched amino acids [147,148,149], uptake of ions (phosphorus [150,151,152], potassium [153,154], molybdenum [155], iron [156,157], sulfur [155], zinc [94] and manganese [158]), nitrate reduction [40], NO modulation [159,160], oxygen limitation responses [40,161,162,163], cell cycle [164,165,166], peptide importers [167,168,169,170,171], regulatory non-coding RNAs (e.g., globally acting trans-small RNA AbcR1/2 and those fragments derived from transfer RNA) [172,173] and carbon–nitrogen metabolism coordination by nitrogen-related phosphotransferase system [153,174,175]. These efforts indicate that the successful integration of key nodulation and nitrogen fixation circuits in various bacterial recipients involves systematic and dynamic coordination with other functions during the establishment of symbiosis.

### 3.2. Integration of Key Symbiosis Circuits with Recipient Regulation Network

Although all key nitrogen-fixing genes are directly activated by NifA in *Proteobacteria* [11], a constitutive expression of nitrogenase should be avoided under fluctuating conditions. The *nifA* gene can be transcriptionally activated by different upstream regulators, e.g., the FixL-FixJ two-component system in *S. meliloti* [176], the FixL-FixJ-FixK cascade in *A. caulinodans* [177,178], the redox responsive regulator RegR and its upstream kinases in *B. diazoefficiens* [162,179,180]. Nitrogenase is O_2_ sensitive and microaerobic fitness machineries seem to be relatively conserved in test rhizobia. The *fixNOQP* operon, encoding the *cbb3* terminal oxidase, is transcriptionally activated by the FixL-FixJ-FixK cascade in *S. meliloti*, *A. caulinodans* and *B. diazoefficiens* [176,177,181,182,183], and the hFixL-FxkR-FixKf-FnrN cascade in *R. etli* and *R. leguminosarum* [163,184]. On the other hand, NifA can activate the transcription of *fixABCX*, encoding an electron bifurcating complex that provides low-potential reducing equivalents for nitrogenase [185], in *S. meliloti*, *R. etli* and *R. leguminosarum* [186,187,188,189]. Ferredoxin, likely reduced by FixABCX during nitrogen fixation [185], is a reductant of nitrogenase and its gene transcription is also activated by NifA in *S. meliloti*, *B. diazoefficiens* and *R. etli* [190,191]. Available transcriptomic evidence in *B. diazoefficiens*, *S. meliloti* and *R. etli* indicates that NifA may regulate more functional genes, e.g., molybdenum transporter, cytochrome P450 proteins, GroES, GroEL and uptake hydrogenase in a strain-dependent manner [161,189,190]. Notably, stringent sets of NifA regulon in these three rhizobia (19–67 genes) are on the symbiosis plasmid or symbiosis island with rare exceptions [161,189,190]. These findings imply that the integration of the key nitrogen fixation gene circuit in terms of transcriptional activation is strain-dependent, and the transcriptional regulation network of bacterial recipients seems to be limitedly subject to direct interference by NifA.

An efficient nitrogen fixation process of rhizobia is structurally ensured by nodule organogenesis that is initiated by specific recognition of host symbiotic signals by NodD in most rhizobia. The divergence of NodD and multiple NodD copies facilitate rhizobia efficiently establishing symbiosis under fluctuating conditions and/or exploring more host plants [64,67,68,192,193]. Available studies support a model where a primary NodD binds DNA in the absence of host symbiotic signals while signal–NodD binding enhances DNA bending that allows transcription [194,195]. NodD autoregulates its own transcription in *R. leguminosarum* bv. *trifolii* and *R. leguminosarum* bv. *viciae* [196,197]. When two or more NodDs are encoded within a genome, these NodDs usually act as a coordination module in a rhizobium- and condition-dependent way. For example, a second NodD copy in some *R. leguminosarum* bv. *trifolii* strains enhances nodule colonization competitiveness [198]. In *S. meliloti*, NodD1*_Sm_* and NodD2*_Sm_* respond to plant signals and the overexpressed NodD3*_Sm_* can function without flavonoids [62,199]. Among five NodD copies of *R. tropici* CIAT899, *nodD2_Rt_* expression is induced by osmotic stress, and the engineered overexpression of NodD2*_Rt_* alone is sufficient to replace the other NodD copies [200]. *Mesorhizobium loti* R7A has two NodD1*_Ml_* and NodD2*_Ml_*, showing a degree of functional redundancy [201]. More detailed investigation demonstrates that NodD1*_Ml_* mainly function in infection threads while NodD2*_Ml_* primarily acts in the rhizosphere and within nodules [202]. This observed division of labor is likely due to their divergence at the signal binding cleft [202]. Noteworthy, NodD2*_Ml_* activity is negatively affected by NodD1*_Ml_* at the pre-infection stage [202]. Two NodD copies are also found in other rhizobia, e.g., *S. fredii* and *B. diazoefficiens*, where NodD2 can negatively regulate the transcription of *nodD1* [193,203,204]. The *nodD2_Sf_* mutant shows impaired nodulation on soybean but improved compatibility with *Lotus* species [205]. Collectively, these findings suggest a working model where rhizobial NodD and its regulon may be divergently selected in at least two dimensions: 1) from rhizosphere to rhizoplane to infection threads to nodules; 2) different host plants. 

Various variables in these niches may interact with the key symbiotic interaction signaling pathway/network. Indeed, NFs are required for the biofilm establishment [206,207], which generally enhances bacterial resilience to various stress factors [208]. The production of exopolysaccharide (EPS), a common component of biofilm matrix, is negatively regulated by NodD in *S. fredii* [65,209] while positively regulated by the NodD3-SyrM-SyrA regulatory module in *S. meliloti* [210]. This is in line with the fact that EPS is an important symbiotic signal for the host infection of *S. meliloti* but dispensable in *S. fredii* symbiosis with test legumes [211,212]. In the presence of symbiotic signal daidzein, *S. fredii* NGR234 produces the phytohormone indole-3-acetic acid (IAA) in a NodD-dependent manner, and overexpression of NodD2*_Sf_* enhances the transcription of IAA synthesis genes and IAA production [213]. The positive regulation of IAA production by NodD is also found in *R. tropici* CIAT 899 [192]. There is evidence showing that flavonoids induce an NodD-dependent expression of *traI* that is responsible for the synthesis of short-chain quorum-sensing 3-oxo-C8-HSL in *S. fredii* [207]. Independent studies also reveal that the transcription of T3SS and effector coding genes depends on the positive regulator TtsI, while the expression of *ttsI* can be activated by NodD and plant flavonoids [214]. In NodD3*_Sm_* over-expressing *S. meliloti*, more than 200 genes are differentially expressed including upregulated EPS biosynthesis, and downregulated motility and chemotaxis functions [215]. Among those upregulated genes (above 70 genes), 69% are located on the chromosome and chromid [215]. Although a systematic survey of direct targets of NodD is not available yet, these examples suggest that NodDs have been evolving to differentially modulate other non-symbiosis functions to improve fitness from rhizosphere to rhizoplane to infection in a strain–host-dependent manner.

In addition to NodD, lineage-specific parts regulating *nod* genes have been identified in a few model rhizobia, e.g., the two-component system NodV-NodW, NwsB and NolA in *B. diazoefficiens* [204], SyrM, a NodD homolog, in *Sinorhizobium* [210,216,217], NolR homologs in *Sinorhizobium* and *Rhizobium* [218,219,220,221,222]. NodV-NodW positively regulates nodulation genes and is essential for symbiosis with mung bean, cowpea and Siratro, but only marginally contributes to nodulation on soybean [223]. NwsB, a homolog of NodW, is required for both induction and the population density-dependent repression of nodulation genes [224]. NolA induces *nodD2_Bd_* and consequently represses the transcription of nodulation genes [225]. SyrM*_Sm_* activates *nodD3_Sm_* transcription and NodD3*_Sm_* induces *syrM_Sm_*, forming a self-amplifying circuit in *S. meliloti* [210]. NodD1_Sf_ induces the transcription of SyrM*_Sf_* that in turn induces NodD2_Sf_ that represses nodulation genes in *S. fredii* [216]. Chromosomal NolR directly targets *nodD1_Sm_*, *nodD2_Sm_* and *nodABC_Sm_* operons in *S. meliloti* [220,226,227] and downregulates nodulation and T3SS genes in *S. fredii* [222].

**Figure 3:**
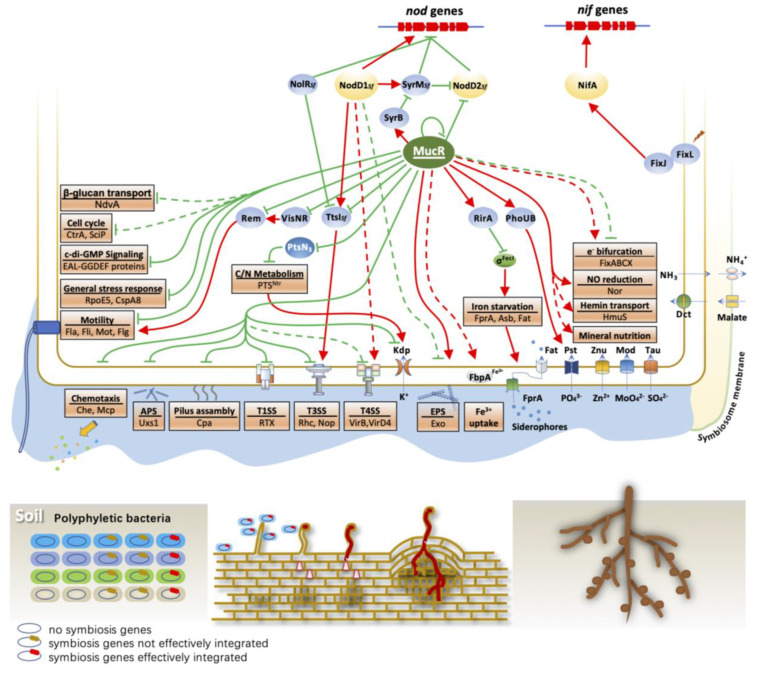
Working model for transcriptional integration of key symbiosis genes. Red arrow, positive regulation; green line, negative regulation. Solid lines indicate cases with molecular evidence. EPS, exopolysaccharide; APS, arabinose-containing polysaccharide; NO, nitric oxide. *_Sf_*, *S. fredii*; *_Sm_*, *S. meliloti*. For simplicity, functional genes in broad-host-range *S. fredii* are shown. The negative regulation of *syrM* by SyrB and positive regulation of *nifA* by FixJ are demonstrated in *S. meliloti*.

MucR/RosR/Ros/MI (hereafter MucR), a conserved zinc-finger regulator in *Alphaproteobacteria*, enhances the induction of key nodulation genes by the host signal luteolin in *S. meliloti* [228]. The disruption of *mucR* leads to delayed nodule development in the *S. meliloti*-alfalfa pair [228], reduced nodulation competitiveness in *R. etli* (common bean) [229], impaired nodulation and nitrogen fixation in *R. leguminosarum* bv. *trifolii* (clover) [230] and deficient nitrogen fixation in broad-host-range *S. fredii* strains (e.g., increased nodule number on soybean while decreased nodule number on *Lotus burttii*) [231,232]. Available transcriptomic analyses independently show that MucR is a pleiotropic regulator, e.g., in *R. leguminosarum* bv. *trifolii*, *S. meliloti*, *S. fredii* and other members of *Alphaproteobacteria* [113,228,231,232,233,234] (Figure 3). MucR autoregulates its transcription and recent ChIP-seq analysis further revealed more than 1350 direct target genes of MucR in the multipartite genome of *S. fredii* [111], among which a considerable number of known circuits were identified, e.g., NodD2_Sf_ [193,203], TtsI, T3SS and its effector NopP [84], SyrB (negative regulator for SyrM in *S. meliloti*) [235], pilus assembly (Cpa), motility (Fla/Fli/Mot/Flg) and chemotaxis (Che/Mcp) [124], diguanylate cyclases [236], general stress response (RpoE5 and CspA8) [237], surface polysaccharides (ExoY and Uxs1) [238,239], carbon–nitrogen metabolism coordination (PtsN3) [153,174], nitric oxide reduction (Nor) [40], and uptake of potassium (Kdp) [153,154], iron (RirA) [156] and phosphorus (PhoUB) [150]. It should be noted that MucR rarely activates its target genes and prefers to bind AT-rich DNA (periodic repeats of “Ts”) that represents a characteristic feature of the symbiosis plasmid, genomic islands and xenogeneic DNA [111]. More molecular biology evidence shows that MucR shares convergently evolved features of xenogeneic silencers with H-NS, Lsr2, MvaT and Rok from either Gram-positive or Gram-negative bacteria [112], i.e., oligomerization mediated by N-terminal domain and forming DNA-protein-DNA bridging complex. The importance of these convergent functions in integrating symbiosis/pathogenesis functions is demonstrated by the H-NS from *E. coli* being interchangeable with MucR from *S. fredii* in the hemolysis activity assay and symbiotic performance [112]. Indeed, H-NS and other functional H-NS/MucR or Lsr2/MucR chimeric proteins, show similar ChIP-seq profiles in the multipartite genome of *S. fredii* and share 286 target genes including those encoding NodD2*_Sf_*, TtsI, T3SS, NopP, RirA, ExoY, Uxs1, TraI, TraR and VisN [112]. This evidence highlights that the convergent xenogeneic silencer MucR predisposes *Alphaproteobacteria* to integrate AT-rich symbiosis genes.

## 4. Conclusions and Perspectives

Rhizobia become sustained in the symbiosis with legume plants due to mutualism, and symbiosis gene circuits become retained in a polyphyletic group of more than 200 bacterial species by conferring symbiotic abilities (Figure 1). This has been proposed as a kind of symbiosis within symbiosis [17]. A similar scenario can be applied to pathogenesis and other transferable or synthesized gene circuits. From this point of view, the process of establishing symbiosis with diverse legumes is just one of the alternative options for soil bacteria. As discussed above, the integration of *nod*/*nif* gene circuits, involving genome innovations and regulation reconstruction (Figure 2 and Figure 3), is not orthogonal in various recipients, which is consistent with observed rhizobial variations in symbiotic performance. Successful integrations of these key symbiosis circuits in diverse recipients should recruit both “global” and “local” regulatory modules, which constitute a regulatory network that is both physiologically and evolutionarily dynamic responding to ever fluctuating niche dimensions (Figure 2 and Figure 3), e.g., conditions of pH and osmolarity, and resources of oxygen, carbon, nitrogen, phosphorus, iron, zinc, potassium, molybdenum, sulfur and manganese. Moreover, the dynamic growth status and evolution of host plants and other surrounding organisms can reconstruct these niche dimensions, in addition to directly exerting biotic influence on rhizobial fitness in saprophytic and symbiotic life cycles. Therefore, an ever-changing realized niche shapes the adaptive evolution of polyphyletic rhizobia after receiving key symbiosis genes.

It is emerging that convergent xenogeneic silencers, e.g., MucR conserved in *Alphaproteobacteria*, may represent one of the most important global regulators in integrating conditional beneficial foreign circuits, including key symbiosis genes [112,113] (Figure 3). MucR mainly downregulates its AT-rich target genes across conservation levels [111] and facilitates insertion mutations in AT-rich conditional deleterious genes by ISs [110]. These silencing mechanisms are crucial for managing adaptive pangenome while maintaining metabolic efficiency. However, we know little about putative anti-silencing mechanisms underlying various adaptive regulons of MucR in different niches, from bulk soils to rhizosphere to rhizoplane to nodules (Figure 3). Moreover, most lineage-specific local regulatory modules have not been systematically investigated on a genome-wide scale using molecular biology techniques (Figure 3), besides available correlation transcriptomic analyses. Apparently, systems biology should be more effectively recruited by scientists to facilitate completing a whole picture of rhizobial adaptive evolution involving ever-rewiring regulation networks. This may support further improvement of rhizobial symbiosis in terms of robustness and efficiency through synthetic biology, which involves rational design and systematic optimization of key symbiosis circuits and their integration efficiency within polyphyletic rhizobia.

## Data Availability

Data sharing not applicable.

## Abbreviations

*nod*	nodulation
*nif*	nitrogen fixation
NFR	nodulation factor receptor
NCR	nodule-specific cysteine-rich
IRLC	inverted repeat lacking clade
SNF	symbiotic nitrogen fixation
HGT	horizontal gene transfer
NF	nodulation factor
T1SS	type I secretion system
T3SS	type III secretion system
T4SS	type IV secretion system
T6SS	type VI secretion system
NO	nitric oxide
IS	insertion sequence
TE	transposable element
EPS	exopolysaccharide
APS	arabinose-containing polysaccharide
IAA	indole-3-acetic acid
ChIP-seq	chromatin immunoprecipitation sequencing
*_Sf_*	*Sinorhizobium fredii*
*_Sm_*	*Sinorhizobium meliloti*
*_Rt_*	*Rhizobium tropici*
*_Ml_*	*Mesorhizobium loti*
*_Bd_*	*Bradyrhizobium diazoefficiens*
